# 2325 Targeting isoform-specific PI3 kinase signaling for treatment of cutaneous T cell lymphoma

**DOI:** 10.1017/cts.2018.128

**Published:** 2018-11-21

**Authors:** Suravi Raychaudhuri, Chi-Heng Wu, Weiyun Ai

**Affiliations:** University of California, San Francisco, CA, USA

## Abstract

OBJECTIVES/SPECIFIC AIMS: (1) Determine the anti-proliferative effect of Copanlisib (α/δ PI3 kinase inhibitor) in CTCL cell lines and synergy with other anti-tumor drugs such as HDAC inhibitors. (2) Determine the effect of Copanlisib treatment on downstream targets of the PI3K/AKT/mTOR pathway. METHODS/STUDY POPULATION: We will test the anti-proliferative effect of Copanlisib on the cell lines H9 and HH, which are well characterized for evaluating new therapies for CTCL (Netchiporouk *et al.*, 2017). We will also test the anti-proliferative effect of Copanlisib in combination with HDAC inhibitors on CTCL cell lines. Cell Titer Glo (Promega) will be used for the proliferation assay. Briefly, Cell-Titler Glo will be added after 24, 48, and 72 hours and luminescence will be measured as a % of maximal growth. Inhibitory effect will be determined by comparison to % growth of the control cultures without Copanlisib treatment. Our next objective is to determine the effect of Copanlisib treatment on downstream targets of the PI3K/AKT/mTOR pathway using Western blot analysis. In brief, 30 μg of each lysate will be subjected to 4%–12% gradient SDS-PAGE gel eletrophoresis. All primary antibodies were purchased from Cell Signaling Technology. Membranes will be washed with TBST and incubated with 1:10,000 dilution of IRDye-conjugated secondary antibody (Licor) for 1 hour. Results will be expressed as relative intensity: the intensity of each band adjusted to that of GAPDH. Experiments will be done in triplicate and 1-way analysis of variance followed by multiple comparison test will be applied to compare the cell proliferation between different treatment groups. RESULTS/ANTICIPATED RESULTS: Previous results from the Ai lab have demonstrated the importance of the PI3 kinase signaling cascade using high-throughput proliferation assays and siRNA knockdown of individual and double PI3 kinase isoforms (unpublished data). So far I have successfully established culture of HH and H9 cells in 96 well plates (100,000 cells/200 μL). We are titrating Copanlisib at doses from 20 nm to 20 μM. Initial results suggest a promising anti-proliferative effect and currently we are optimizing the most effective pharmacologic dose. Thus we anticipate that Copanlisib will exhibit a potent anti-proliferation activity in CTCL cell lines. H9 and HH cell lysates have been collected and preserved for Western blot analysis. We anticipate that Copanlisib treatment will significantly decrease the phosphorylation of AKT and 4EB-P1, both downstream targets of PI3 kinase signaling. DISCUSSION/SIGNIFICANCE OF IMPACT: These findings will elucidate the importance of the PI3 kinase/AKT/mTOR pathway in tumor proliferation in CTCL. Identifying the importance of specific isoforms of PI3 kinase in CTCL will allow for more targeted selection of treatment. Copanlisib targets α and δ isoforms of PI3K and is newly approved by the FDA for low grade B cell lymphoma. Our results seek to quantify the anti-proliferative effect of Copanlisib and determine an on-target mechanism of action by investigating the drug’s effects on downstream signaling molecules of the PI3 kinase pathway. This project will elucidate the disease process of CTCL and provide important insight for its management.

